# Validation of a deep-learning segmentation model for adult and pediatric head and neck radiotherapy in different patient positions

**DOI:** 10.1016/j.phro.2023.100527

**Published:** 2023-12-27

**Authors:** Linda Chen, Patricia Platzer, Christian Reschl, Mansure Schafasand, Ankita Nachankar, Christoph Lukas Hajdusich, Peter Kuess, Markus Stock, Steven Habraken, Antonio Carlino

**Affiliations:** a MedAustron Ion Therapy Center, Department of Medical Physics, Wiener Neustadt, Austria; b Erasmus MC Cancer Institute, University Medical Center, Department of Radiotherapy, Rotterdam, the Netherlands; c Delft University of Technology, Faculty of Mechanical, Maritime and Materials Engineering, Delft, the Netherlands; d Leiden University Medical Center, Faculty of Medicine, Leiden, the Netherlands; e Fachhochschule Wiener Neustadt, Department MedTech, Wiener Neustadt, Austria; f Medical University of Vienna, Department of Radiation Oncology, Vienna, Austria; g ACMIT Gmbh, Department of Medicine, Wiener Neustadt, Austria; h Karl Landsteiner University of Health Sciences, Department of Oncology, Krems an der Donau, Austria; i Holland Proton Therapy Center, Department of Medical Physics & Informatics, Delft, the Netherlands

**Keywords:** Autocontouring, Radiation therapy, Artificial Intelligence, Head and neck cancer, Auto-segmentation, Organs-at-risk

## Abstract

**Background and purpose:**

Autocontouring for radiotherapy has the potential to significantly save time and reduce interobserver variability. We aimed to assess the performance of a commercial autocontouring model for head and neck (H&N) patients in eight orientations relevant to particle therapy with fixed beam lines, focusing on validation and implementation for routine clinical use.

**Materials and methods:**

Autocontouring was performed on sixteen organs at risk (OARs) for 98 adult and pediatric patients with 137 H&N CT scans in eight orientations. A geometric comparison of the autocontours and manual segmentations was performed using the Hausdorff Distance 95th percentile, Dice Similarity Coefficient (DSC) and surface DSC and compared to interobserver variability where available. Additional qualitative scoring and dose-volume-histogram (DVH) parameters analyses were performed for twenty patients in two positions, consisting of scoring on a 0–3 scale based on clinical usability and comparing the mean (D_mean_) and near-maximum (D_2%_) dose, respectively.

**Results:**

For the geometric analysis, the model performance in head-first-supine straight and hyperextended orientations was in the same range as the interobserver variability. HD95, DSC and surface DSC was heterogeneous in other orientations. No significant geometric differences were found between pediatric and adult autocontours. The qualitative scoring yielded a median score of ≥ 2 for 13/16 OARs while 7/32 DVH parameters were significantly different.

**Conclusions:**

For head-first-supine straight and hyperextended scans, we found that 13/16 OAR autocontours were suited for use in daily clinical practice and subsequently implemented. Further development is needed for other patient orientations before implementation.

## Highlights

• The use of deep learning-generated contours is feasible for organs at risk in head and neck radiotherapy.
• When the model was given patient scans in other orientations than the model was trained on, the autocontours were not suitable for use in the clinic, showing the need for validation of models in the local setting.
• The autocontours for 13/16 studied structures could be used in the clinic from a geometric, dose/volume and qualitative perspective for patients in head-first-supine straight and hyperextended position.

## 1 Introduction

Radiation therapy (RT) is one of the pillars of treatment for tumors in the head and neck (H&N) [1]. Accurate delineation of the tumor and the organs at risk (OARs) is essential for a safe and optimal treatment for patients. However, manual delineation in the head and neck region is a labor-intensive process and is associated with high inter-observer variability (IOV) [2], [3], [4], [5].

For this reason, automated solutions are appealing. Thresholding, atlas-based, (mechanical) model-based or machine learning-based solutions enable some of the labor to be transferred to the computer [6]. The use of these algorithms enables faster delineations of OARs and reduces IOV, leading to improvements in quality and consistency [7], [8]. Deep learning (DL)-based contouring models were found to be feasible for clinical use and found significant time reduction [9], [10].

That being said, conventional photon therapy makes use of gantries, allowing the vast majority of patients to be treated in head-first-supine (HFS) position. However, there are also centers that make use of fixed beam lines, such as dual-particle radiotherapy facilities treating patients with carbon-ions and protons [11]. Consequently, a larger variety of patient orientations is required to mimic ideal beam angles and minimize OAR toxicity [12], [13]. As patients at particle centers are treated in various orientations, it is crucial to validate existing autocontouring models on center-specific data in various orientations before introducing autocontouring to clinical practice.

In this study, we aimed to assess the performance of the commercial automatic segmentation model for adult and pediatric H&N patients in eight different orientations from a geometric, dose/volume and qualitative perspective, focusing on the implementation for routine clinical use.

## 2 Materials and methods

### 2.1 Study design and data collection

The retrospective study was conducted at MedAustron, using the RSL RayMachine deep-learning algorithm for auto-contouring (v2.0.0.45) in H&N patients that was integrated in RayStation v11B. The study was approved by the review board of Lower Austria (GS1-EK-4/350–2015).

The training involved neural networks of 3D CNN U-net type, performed by RaySearch Laboratories AB (RSL). Each network was trained using supervised learning on annotated CT scans of male and female adult H&N patients, including patients with dental artefacts. All patients were in HFS straight orientation. The input dimensions and resolutions varied based on the characteristics of the OARs each network was supposed to segment. The training data was augmented using rotations (random rotation of 0–5 degrees around all axes), translations, and elastic deformations. The DL result was post-processed to smooth surfaces, remove noise and resolve overlaps between structures. A structure was only returned when the model localized the OAR.

The dataset consisted of 98 patients with 137 anonymized planning computed tomography (CT) scans who were treated at MedAustron with proton or carbon ion RT between January 2020 and September 2022. One to three CT scans were acquired for each patient, as some patients needed to be treated in several orientations to reduce the risk of toxicity. In our patient population, 60 patients had tumors in the central nervous system (CNS) and 38 patients in the H&N region, providing 83 and 54 CT scans, respectively. CTs were acquired with the Philips Brilliant BigBore Oncology CT (Philips Healthcare, Andover, USA) applying the clinical protocol (slice thickness 2 mm for CNS/3 mm for H&N, FOV 350 mm, 120 kV, 300 mAs/slice, helical) without contrast agent. Head-step immobilization (5-point mask) was used for 67 CT scans and base of skull immobilization (3-point mask) for 70 CT scans.

Demographic data, parameters relevant to RT planning, and immobilization data are summarized in Table 1. The patients’ age ranged from 1 to 79 years (median: 45 years). The study consisted of 48 % male and 52 % female participants. We collected 95 scans from adult patients and 42 scans from pediatric patients. CT scans of seven patients included a bite positioner or intubation equipment. The available scans were divided into eight categories according to the patients’ orientation: 1) HFS straight; 2) HFS hyperextended neck; 3) head-first-decubitus (HFD) left; 4) HFD right; 5) HFS rotated left; 6) HFS rotated right; 7) head-first-prone (HFP) left; 8) HFP right (Table 1).

**Table 1:** Baseline data of study population and patient orientations.

Characteristic			Study population
Sex (male, %)			47 (48 %)
Age (median, range, yr)			45 [1–79]
Age categories (n)			
Adult			72
Pediatric			26

Patient orientations	Diagnostic group		Number of CT scans (%)
	CNS (n, %)	HNC (n, %)	

Category 1: HFS Straight	25 (18 %)	6 (4 %)	31 (23 %)
Category 2: HFS Hyperextension	12 (9 %)	22 (16 %)	34 (25 %)
Category 3: HFD left	9 (7 %)	3 (2 %)	12 (9 %)
Category 4: HFD right	15 (11 %)	2 (1 %)	17 (12 %)
Category 5: HFS Rotated left	2 (1 %)	14 (10 %)	16 (12 %)
Category 6: HFS Rotated right	10 (7 %)	7 (5 %)	17 (12 %)
Category 7: HFP left	7 (5 %)	0 (0 %)	7 (5 %)
Category 8: HFP right	3 (2 %)	0 (0 %)	3 (2 %)
**Total**	**83 (61 %)**	**54 (39 %)**	**137 (100 %)**

CNS = central nervous system; HNC = head and neck cancer;. HFS = head-first-supine; HFD = head-first-decubitus; HFP = head-first-prone.

We considered overlapping OARs from the RSL model and the clinical H&N protocol, which were the following sixteen OARs according to standard delineation guidelines [14], [15], [16], [17]: mandible; brain; brainstem; oral cavity; eye (left); eye (right); lacrimal gland (left); lacrimal gland (right); lens (left); lens (right); optic chiasm; optic nerve (left); optic nerve (right); parotid (left); parotid (right); spinal cord.

Manual segmentations were made by radiation oncologists and radiation technologists on the treatment planning system RSL RayStation v8B and v11B. To perform the study, planning CTs, registrations, planning magnetic resonance imaging (MRIs), manual structure sets, and treatment plans were obtained and anonymized. Evaluation of the model performance was performed using Python 3.7 in the RayStation scripting environment.

### 2.2 Geometric analysis

We geometrically evaluated the model using the Hausdorff Distance 95th percentile (HD95), Dice Similarity Coefficient (DSC) and surface DSC at 2 mm (sDSC) to allow for comparison with similar studies [7], [8], [18]. The DSC quantifies the overlap of the manual contour and the autocontours, whilst the sDSC provides information on the overlap of the surfaces of the two contours at 2 mm [19], [20]. As the DSC decreases rapidly in small structures, the HD95 provides useful additional information about the general location of the autocontour as well as evaluating the edges of the manual contours and autocontours [21].

For each OAR, the mean and standard deviation of the DSC, HD95 and sDSC was calculated for each category. DSC and HD95 were compared to the interobserver variability (IOV) values from previous studies when possible [4], [5]. We compared DSC, HD95 and sDSC in adult and pediatric scans, and in HFS straight and HFS hyperextended orientations using the Mann-Whitney *U* test for all OARs that had three or more samples [22]. Threshold for significance was set at 0.05 divided by number of analyzed OARs as per Bonferroni correction for multiple testing [23].

### 2.3 Qualitative scoring

For the qualitative scoring and dose/volume analysis, a representative subset was selected to reduce the workload of the analyses. Twenty scans from different patients were selected, which were from HFS straight and HFS hyperextended neck orientations, as data from other orientations was too heterogeneous to analyze further.

Two reviewers, both radiation technologists, responsible for OAR delineation in clinical practice and with seven years of experience, performed the qualitative assessment using the planning CT with the autocontours, with the planning MRIs as an optional aid. All OARs were scored on the following scale [7]: 0) not acceptable, complete re-drawing needed, no time-gain; 1) major corrections needed, but model still useful, minor time gain; 2) minor corrections needed, significant time gain; 3) accepted without corrections, significant time gain.

To determine the agreement between observers, the intraclass correlation (ICC) was calculated [24], [26]. OARs with a median value of two or higher were considered for implementation in clinical practice. Qualitative scoring for patient positioning in HFS straight and hyperextended neck were compared.

### 2.4 Dose/volume analysis

To assess the feasibility of autocontouring in a clinical work flow in terms of dose and dose-to-volume, the dataset for the qualitative scoring was used. The DVH parameters for each patient were recalculated with the automatic contours using the existing treatment plans to isolate the effect of the contours on the doses. For this, the Monte Carlo algorithm v5.3 for protons and pencil beam algorithm v4.4 (radiobiological model LEM 1) for carbon ions developed by RSL was used. Treatment plans were composed of three to five beams depending on the prescribed dose and the beam arrangement was adapted to the specific tumor location and OARs constraints with a minimum spacing in between of thirty degrees. The studied OARs were included in the treatment plan optimization if they were in proximity of the treated volume. The DVH parameters D_2%_ and D_mean_ were compared for the automatic and manual contours using the Wilcoxon signed-rank test [25]. DVH parameters for patient positioning in HFS straight and hyperextended neck were compared. Threshold for significance was set at 0.05 divided by number of analyzed OARs as per Bonferroni correction for multiple testing [23].

## 3 Results

**Fig. 1:**
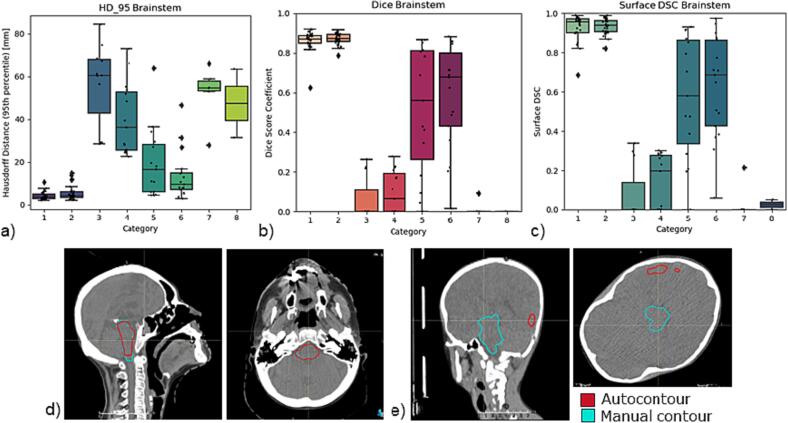
Geometric analysis of the brainstem. a) Hausdorff Distance 95th percentile (HD95) for the brainstem. Categories 1 and 2 show results similar to interobserver variability (dashed line). The other categories vary in performance. b) Dice Similarity Coefficient (DSC) for the brainstem. c) Surface DSC (3 mm) for the brainstem. d) Example brainstem segmentation of a scan in hyperextended position with reasonable agreement between the manual contour (blue) and the autocontour (red). e) Example brainstem segmentation of a scan in prone position with poor agreement between the manual contour (blue) and the autocontour (red). (For interpretation of the references to colour in this figure legend, the reader is referred to the web version of this article.)

**Fig. 2:**
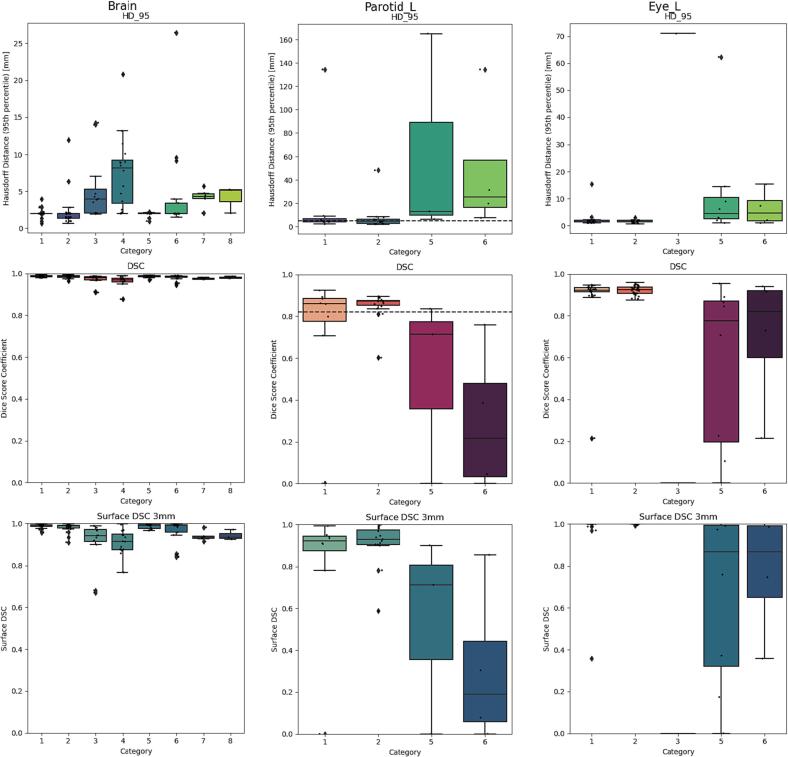
Geometric analysis for the brain, left parotid and left eye with the interobserver variability (dashed line) when available. The upper row displays the Hausdorff Distance 95th percentile (HD95), the middle row the Dice Similarity Coefficient (DSC) and the lower row the Surface DSC. Not all categories are displayed, as the model only delivers autocontours for organs at risk that it is able to localize.

For the geometric analysis of the brainstem, the HD95 was 4.2 ± 1.9 mm and 7.5 ± 12.0 mm (IOV HD = 4.0 mm), DSC was 0.9 ± 0.1 and 0.8 ± 0.1 (IOV DSC = 0.88) and sDSC was 0.9 ± 0.1 and 0.9 ± 0.0 in HFS straight and hyperextended groups, respectively (Fig. 1*,* Supplementary Data *A*). For HFD left and HFP left, HD95 was 56.4 ± 18.4 mm and 52.5 ± 11.8 mm, and DSC and sDSC were 0.1 ± 0.1 and 0.0 ± 0.0 in the brainstem. For the left parotid, the HD95 was 20.9 ± 42.9 mm and 7.4 ± 11.1 mm, DSC was 0.7 ± 0.3 and 0.9 ± 0.1 (IOV DSC = 0.82) and the sDSC was 0.8 ± 0.3 and 0.9 ± 0.1 in the HFS straight and hyperextended group, respectively. In contrast, HD95 was 61.5 ± 73.3 mm and 48.2 ± 50.4 mm, and DSC and sDSC were 0.5 ± 0.4 and 0.3 ± 0.3 for the left parotid in HFS Rotated left and HFS Rotated right, respectively. Fig. 2 shows the HD95 and DSC for the brain, the left eye, left parotid with the corresponding IOV for each category, with maximum HD95 of 71.1 mm and minimum DSC and sDSC of 0.

The following eleven OARs had three or more samples for comparison between adult and pediatric scans: brain; brainstem; eye (left); eye (right); lens (left); lens (right); optic chiasm; optic nerve (left); optic nerve (right); parotid (left); parotid (right). No OARs were significantly different in the DSC, HD95 or sDSC between the adult and pediatric groups or HFS straight and hyperextended groups (Supplementary data *B* and Supplementary data *C*, respectively).

**Fig. 3:**
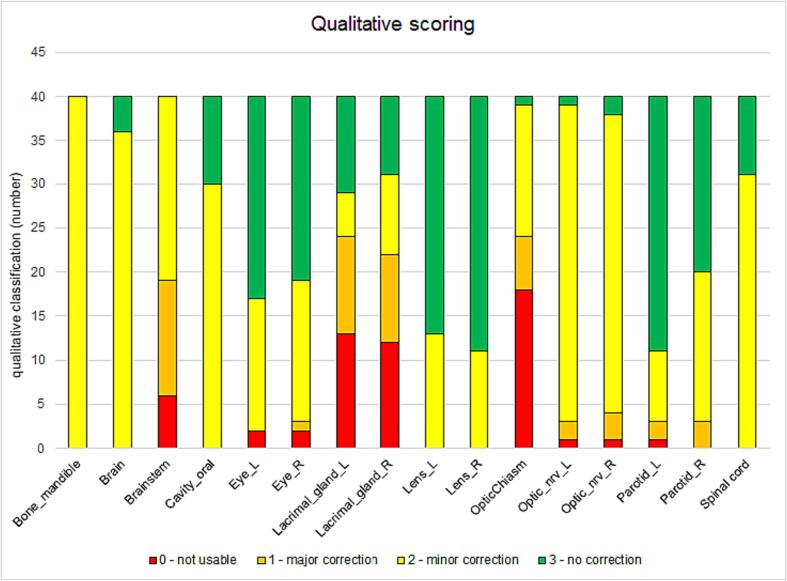
Distribution of scores for all regions of interest (ROIs) in the qualitative analysis dataset of twenty scans in HFS traight and hyperextended orientations. The median score for each ROI is displayed on top of the bar. L = left, R = right.

For the qualitative analysis, the observers report an ICC of 0.64, signifying moderate agreement. Overall, thirteen out of sixteen (81%) of the OARs had a median ≥ 2, which would not be changed by the exclusion of the HFS hyperextended position group (Supplementary data *D*). Overall, the mandible, brain, brainstem, oral cavity, left and right optic nerve, right parotid and spinal cord had a median score of two (Fig. 3). The left and right eye, the left and right lens, and the left parotid had a median score of three. The left and right lacrimal gland and the optic chiasm had a median score of one.

**Fig. 4:**
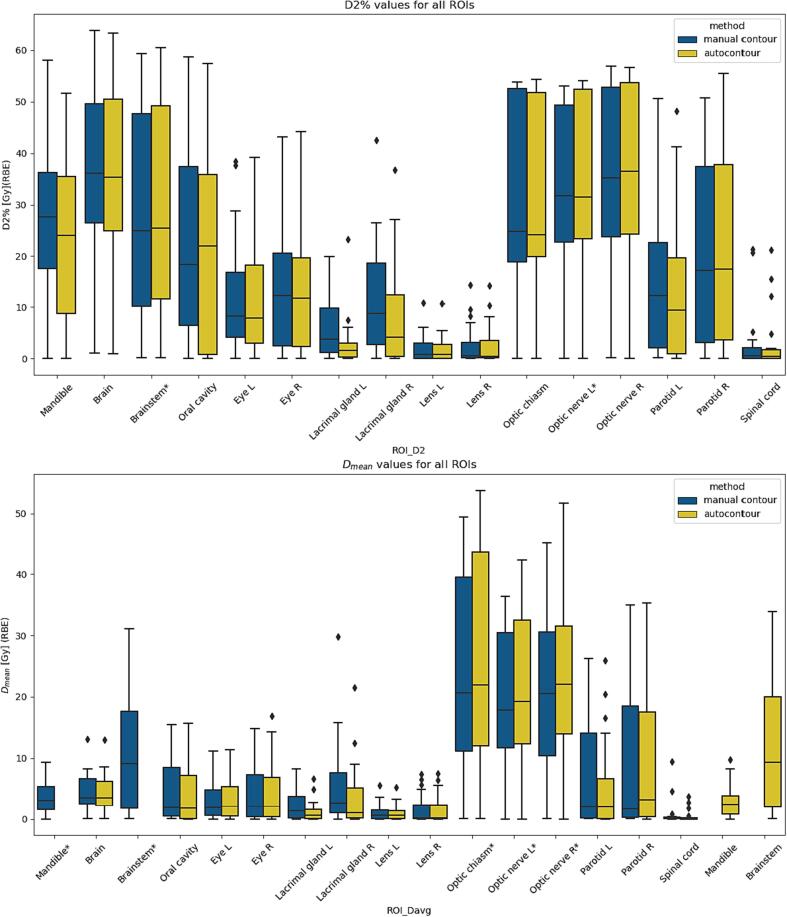
Boxplots of all regions of interest (ROIs) for manual and autocontours for D_2%_ (a) and D_mean_ (b). Asterisk (*) denotes a significant different between manual contours and autocontours. L = left, R = right.

Seven out of thirty-two DVH parameters were significantly different: D_2%_ was significantly different in two out of sixteen OARs and D_mean_ was significantly different in five out of sixteen OARs (Fig. 4). The median D_2%_ was higher in the manual contours for the optic nerve (L) (31.7 vs. 31.6 Gy (RBE), p < 0.01) and higher for the autocontours than the manual contours for the brainstem (25.5 vs. 24.9 Gy (RBE), p < 0.01) (Supplementary data *E*). For the median D_mean_, the dose for the manual contours was higher in the mandible (3.0 vs 2.4 Gy (RBE), p < 0.01). The median D_mean_ was higher for the autocontours in the brainstem (9.1 vs. 9.4 Gy (RBE), p < 0.01), optic chiasm (20.7 vs. 21.9 Gy (RBE), p < 0.01), optic nerve (L) (17.8 vs. 19.3 Gy (RBE), p < 0.01), and optic nerve (R) (20.5 vs. 22.0 Gy (RBE), p < 0.01). No DVH parameters were significantly different between the HFS straight and hyperextended group (Supplementary data *F*).

## 4 Discussion

In this study, we assessed the performance of a commercial automatic segmentation model for H&N patients in various patient treatment orientations from a geometric, dose/volume and qualitative perspective, focusing on the validation and implementation for routine clinical use. Our main findings are that from the eight patient orientations, only the autocontours created for HFS straight and hyperextended orientations were feasible for use in clinical practice without major corrections, as was confirmed by the dose/volume and qualitative analysis for thirteen out of sixteen OARs.

Our results for the geometric analysis varied substantially among the different positioning categories. The largest cause for erroneous segmentation was due to the localization submodel of the algorithm. The model only returned autocontours if it had localized the OAR, resulting in a different number of autocontours for each OAR and did not guarantee a correct localization. Moreover, there were cases of erroneous localization in the defined autocontours. We observed that the location of some autocontours was entirely incorrect when the patient orientation was unexpected to the model. OARs such as the brain, the lens and the eyes were generally well segmented in HFS straight and hyperextended orientations, likely due to the predictability of the shape and contrast differences at the edges. That being said, these OARs were still prone to errors such as left–right confusion, e.g. in decubitus orientation. Moreover, if OARs did not have the expected shape, the model failed to segment the structure accurately, such as the oral cavity if the patient had a bite positioner or was intubated. In these cases, the autocontours still had the expected shape of the OAR in HFS straight position.

Structures for which manual segmentation was heavily reliant on MRI, such as the brainstem or the parotids were generally well segmented in HFS straight and hyperextended orientations. Overall, the results of this study in the HFS straight and hyperextended groups are in line with similar studies in literature [27], [28], [29], [30]. Ayyalusamy et al. analyzed the influence of patient positioning on the performance of an atlas-based segmentation system with HFS straight and hyperextended orientations [31]. They found that improved anatomy matching resulted in better segmentation, and the brainstem was found to be less dependent on head position, which is in line with our findings for the brainstem segmentation for HFS straight and hyperextended orientations [31]. Men et al. demonstrated that the orientation of the training dataset affects segmentation in rectal cancer patients in supine and prone position, with the model accuracy benefitting from being trained on patients in several positioning orientations [32].

Extending the applicability of the model to pediatric patient scans would be highly beneficial. However, as the intended use of the model was for adult patient scans, it was necessary to investigate the presence of significant geometric differences between adult and pediatric scans. We found that none of the compared OARs were significantly different, which indicates that the model is able to adapt to the different proportions found in adult and pediatric H&N regions. This finding greatly increases the applicability of the model and potential time saved in clinical practice.

The OARs for which we found significant differences in the dose/volume analysis corresponded overall with the model performance in the geometric analysis. The analysis of dose/volume parameters was limited to D_2%_ and D_mean_, as these parameters are typically used as clinical constraints [33], [34]. Structures like the optic nerves were significantly different in the DVH parameters, which is in line with the geometric analysis results. Also, although seven DVH parameters differed significantly between manual contours and autocontours, these differences had limited clinical relevance as the absolute dose differences were limited. Thus, the results of the DVH parameter analysis did not dictate the implementation in clinical practice.

Our qualitative analysis showed that 81% of the OARs needed none or only minor corrections. Again, the OARs that performed well corresponded overall with the geometrical and dose/volume analyses. These results are indicative of a benefit in clinical practice, though it should be noted that this analysis is subjective. Despite the subjectivity of the qualitative analysis, it reflects the potential time gain and motivation to use the DL model, which is why we decided to base our choice for clinical implementation on this analysis. We have successfully implemented the thirteen OARs needing no or minor corrections, which has led to up to 20 % of time reduction for delineation per patient.

This study had several limitations. Firstly, it should be noted that we did not replan the treatment plan using the autocontours but used the existing treatment plan for the manual contours and autocontours, similar to a study by Gan et al. [35]. This way, we isolated the effect of the autocontours. However, to fully evaluate the clinical feasibility and to test the autocontours in the clinical workflow, full replanning with the autocontours could be considered for future studies [8].

Another limitation of the study was that the manual delineations had already been made, so it was not possible to directly quantify the time advantage of the model usage, a problem that has been described earlier [10], [36]. For a future study, this quantification could be of added value. Moreover, taking the time into account that is needed for manual adjustments such as in the study by van Dijk et al. would give more insight in the clinical applicability of the DL model [37].

As the model performance in the positioning categories apart from HFS straight and hyperextended varied widely, we only performed the two additional analyses on CT scans in HFS straight and hyperextended orientations. To improve the deep learning segmentation model, improvement of the localization submodel is crucial. A method for this would be retraining with more challenging CT scans in various orientations and with immobilization equipment such as the bite positioner. Moreover, expanding the training data augmentation or more structural improvements in the localization submodel could improve model performance. Furthermore, contouring of the brainstem, lacrimal glands, optic nerves and optic chiasm is largely reliant on MRI scans in clinical practice, and MRI is also heavily used in the contouring of the parotids, pituitary and spinal cord [38]. Because of this, to improve the model performance, integrating MRI scans into the model is likely to be of added value. Considering incorporating other technology like synthetic MRIs, interactive DL models or transfer learning might lead to better results in a heterogeneous dataset such as ours [18], [32], [39], [40]. Allowing manual rotation before segmentation by creating several models for different orientations could also improve performance.

All in all, the commercial deep-learning segmentation model for head and neck showed promising results for HFS straight and hyperextended scans. For other orientations, the model in its current form cannot be used without major modifications of the autocontours in clinical practice. For HFS straight and hyperextended scans, the autocontours of mandible, brain, brainstem, oral cavity, eyes, lenses, and optic nerves, parotids, and spinal cord were deemed clinically suitable from geometric, dose/volume, and qualitative perspectives.

## CRediT authorship contribution statement

**Linda Chen:** Methodology, Investigation, Software, Formal analysis, Writing – original draft, Visualization, Data curation. **Patricia Platzer:** Data curation. **Christian Reschl:** Data curation, Investigation. **Mansure Schafasand:** Writing – review & editing, Software. **Ankita Nachankar:** Data curation. **Christoph Lukas Hajdusich:** Data curation. **Peter Kuess:** Writing – review & editing, Supervision. **Markus Stock:** Conceptualization, Writing – review & editing, Supervision, Resources. **Steven Habraken:** Methodology, Writing – review & editing, Supervision. **Antonio Carlino:** Conceptualization, Methodology, Writing – review & editing, Supervision.

## Declaration of competing interest

The authors declare that they have no known competing financial interests or personal relationships that could have appeared to influence the work reported in this paper.
