# Supplementary material for: Validation of a deep-learning segmentation model for adult and pediatric head and neck radiotherapy in different patient positions
Source: Phys Imaging Radiat Oncol. 2023 Dec 27;29:100527. doi: 10.1016/j.phro.2023.100527 (PMC10787237; doi:10.1016/j.phro.2023.100527)
Supplement: Supplementary data 1 [file mmc1.docx]

**Supplementary data A**

*Table with Hausdorff Distance 95 (HD95) [mm], Dice Similarity Coefficient (DSC) and surface DSC (sDSC) for all organs at risk (OARs), with the reference interobserver variability (IOV) if available and the number of scans available per OAR. L = left, R = right, HFS = head-first-supine, HFD = head-first-decubitus, HFP = head-first-prone. The deep learning segmentation model only returned autocontours if it successfully localizes the OAR.*

| **OAR** | **Mandible** | | | | **Brain** | | | | **Brainstem** | | | | **Oral cavity** | | | |
| --- | --- | --- | --- | --- | --- | --- | --- | --- | --- | --- | --- | --- | --- | --- | --- | --- |
| **Category** | **HD95** | **DSC** | **sDSC** | **n (%)** | **HD95** | **DSC** | **sDSC** | **n (%)** | **HD95** | **DSC** | **sDSC** | **n (%)** | **HD95** | **DSC** | **sDSC** | **n (%)** |
| **1: HFS straight (n=31)** | 10.1 ± 3.0 | 0.8 ± 0.0 | 0.9 ± 0.0 | 13 (42%) | 2.0 ± 0.5 | 1.0 ± 0.0 | 1.0 ± 0.0 | 30 (97%) | 4.2 ± 1.9 | 0.9 ± 0.1 | 0.9 ± 0.1 | 30 (97%) | 17.0 ± 7.3 | 0.7 ± 0.1 | 0.6 ± 0.1 | 8 (26%) |
| **2: HFS hyperextended (n=34)** | 7.1 ± 2.8 | 0.8 ± 0.1 | 0.9 ± 0.0 | 17 (50%) | 2.2 ± 2.0 | 1.0 ± 0.0 | 1.0 ± 0.0 | 31 (91%) | 7.5 ± 12.0 | 0.8 ± 0.1 | 0.9 ± 0.0 | 33 (97%) | 13.4 ± 7.5 | 0.8 ± 0.1 | 0.7 ± 0.2 | 9 (26%) |
| **3: HFD left (n=12)** | - | - | - | 0 (0%) | 5.3 ± 4.2 | 1.0 ± 0.0 | 0.9 ± 0.1 | 12 (100%) | 56.4 ± 18.4 | 0.1 ± 0.1 | 0.1 ± 0.1 | 11 (92%) | - | - | - | 0 (0%) |
| **4: HFS right (n=17)** | - | - | - | 0 (0%) | 7.5 ± 4.9 | 1.0 ± 0.0 | 0.9 ± 0.1 | 16 (94%) | 35.6 ± 15.9 | 0.2 ± 0.2 | 0.1 ± 0.1 | 15 (88%) | - | - | - | 0 (0%) |
| **5: HFS Rotated left (n=16)** | 14.6 ± 9.8 | 0.8 ± 0.1 | 0.9 ± 0.1 | 10 (63%) | 1.9 ± 0.3 | 1.0 ± 0.0 | 1.0 ± 0.0 | 15 (94%) | 19.5 ± 16.0 | 0.5 ± 0.3 | 0.6 ± 0.3 | 15 (94%) | 19.3 ± 6.8 | 0.7 ± 0.0 | 0.5 ± 0.0 | 3 (19%) |
| **6: HFS Rotated right (n=17)** | 17.8 ± 5.9 | 0.8 ± 0.1 | 0.8 ± 0.1 | 3 (18%) | 4.5 ± 6.0 | 1.0 ± 0.0 | 1.0 ± 0.1 | 17 (100%) | 13.7 ± 11.1 | 0.6 ± 0.3 | 0.6 ± 0.3 | 17 (100%) | 16.2 ± 7.2 | 0.6 ± 0.2 | 0.5 ± 0.2 | 3 (18%) |
| **7: HFP left (n=7)** | - | - | - | 0 (0%) | 4.2 ± 1.1 | 1.0 ± 0.0 | 0.9 ± 0.0 | 6 (86%) | 52.5 ± 11.8 | 0.0 ± 0.0 | 0.0 ± 0.0 | 6 (86%) | - | - | - | 0 (0%) |
| **8: HFP right (n=3)** | - | - | - | 0 (0%) | 4.2 ± 1.5 | 1.0 ± 0.0 | 0.9 ± 0.0 | 3 (100%) | 47.5 ± 16.0 | 0.00 ± 0.0 | 0.0 ± 0.0 | 2 (67%) | - | - | - | 0 (0%) |
| **IOV** | 3.4 | 0.9 | - | - | - | - | - |  | 4.0 | 0.9 | - |  | 14.5 | 0.8 | - |  |
| **No of contours compared** | 43 | | | | 130 | | | | 129 | | | | 23 | | | |

| **OAR** | **Eye L** | | | | **Eye R** | | | | **Lacrimal gland L** | | | | **Lacrimal gland R** | | | |
| --- | --- | --- | --- | --- | --- | --- | --- | --- | --- | --- | --- | --- | --- | --- | --- | --- |
| **Category** | **HD95** | **DSC** | **sDSC** | **n (%)** | **HD95** | **DSC** | **sDSC** | **n (%)** | **HD95** | **DSC** | **sDSC** | **n (%)** | **HD95** | **DSC** | **sDSC** | **n (%)** |
| **1: HFS straight (n=31)** | 2.2 ± 2.6 | 0.9 ± 0.1 | 1.0 ± 0.1 | 28 (90%) | 3.8 ± 11.1 | 0.9 ± 0.2 | 1.0 ± 0.2 | 29 (94%) | 8.7 ± 4.3 | 0.2 ± 0.2 | 0.6 ± 0.3 | 18 (58%) | 8.0 ± 4.4 | 0.3 ± 0.2 | 0.6 ± 0.3 | 17 (55%) |
| **2: HFS hyperextended (n=34)** | 1.7 ± 0.5 | 0.9 ± 0.0 | 1.0 ± 0.0 | 33 (97%) | 1.7 ± 0.7 | 0.9 ± 0.0 | 1.0 ± 0.0 | 33 (97%) | 8.1 ± 4.5 | 0.3 ± 0.2 | 0.6 ± 0.3 | 21 (62%) | 8.6 ± 4.5 | 0.4 ± 0.2 | 0.7 ± 0.2 | 19 (56%) |
| **3: HFD left (n=12)** | 71.1 ± 0.0 | 0.0 ± 0.0 | 0.0 ± 0.0 | 1 (8%) | 56.1 ± 0.0 | 0.0 ± 0.0 | 0.0 ± 0.0 | 1 (8%) | - | - | - | 0 (0%) | - | - | - | 0 (0%) |
| **4: HFS right (n=17)** | - | - | - | 0 (0%) | 34.4 ± 23.1 | 0.3 ± 0.4 | 0.0 ± 0.0 | 2 (12%) | - | - | - | 0 (0%) | - | - | - | 0 (0%) |
| **5: HFS Rotated left (n=16)** | 12.6 ± 19.3 | 0.6 ± 0.4 | 0.7 ± 0.4 | 8 (50%) | 4.1 ± 3.2 | 0.8 ± 0.2 | 0.9 ± 0.2 | 14 (88%) | 38.8 ± 28.9 | 0.0 ± 0.00 | 0.1 ± 0.1 | 2 (13%) | 8.9 ± 1.6 | 0.1 ± 0.1 | 0.4 ± 0.2 | 3 (19%) |
| **6: HFS Rotated right (n=17)** | 6.4 ± 5.7 | 0.7 ± 0.3 | 0.8 ± 0.3 | 4 (24%) | 16.6 ± 23.2 | 0.6 ± 0.4 | 0.6 ± 0.4 | 5 (29%) | 15.4 ± 0.0 | 0.0 ± 0.0 | 0.2 ± 0.0 | 1 (6%) | - | - | - | 0 (0%) |
| **7: HFP left (n=7)** | - | - | - | 0 (0%) | - | - | - | 0 (0%) | - | - | - | 0 (0%) | - | - | - | 0 (0%) |
| **8: HFP right (n=3)** | - | - | - | 0 (0%) | - | - | - | 0 (0%) | - | - | - | 0 (0%) | - | - | - | 0 (0%) |
| **IOV** | - | - | - |  | - | - | - |  | - | - |  |  | - | - |  |  |
| **No of contours compared** | 74 | | | | 84 | | | | 42 | | | | 39 | | | |

| **OAR** | **Lens L** | | | | **Lens R** | | | | **Optic chiasm** | | | | **Optic nerve L** | | | |
| --- | --- | --- | --- | --- | --- | --- | --- | --- | --- | --- | --- | --- | --- | --- | --- | --- |
| **Category** | **HD95** | **DSC** | **sDSC** | **n (%)** | **HD95** | **DSC** | **sDSC** | **n (%)** | **HD95** | **DSC** | **sDSC** | **n (%)** | **HD95** | **DSC** | **sDSC** | **n (%)** |
| **1: HFS straight (n=31)** | 1.4 ± 0.7 | 0.8 ± 0.1 | 1.0 ± 0.0 | 23 (74%) | 1.4 ± 1.3 | 0.8 ± 0.2 | 1.0 ± 0.1 | 22 (71%) | 8.8 ± 4.2 | 0.4 ± 0.2 | 0.7 ± 0.2 | 24 (77%) | 3.0 ± 1.5 | 0.7 ± 0.1 | 1.0 ± 0.0 | 28 (90%) |
| **2: HFS hyperextended (n=34)** | 1.3 ± 0.6 | 0.8 ± 0.1 | 1.0 ± 0.0 | 29 (85%) | 1.2 ± 0.6 | 0.8 ± 0.1 | 1.0 ± 0.0 | 29 (85%) | 8.7 ± 2.4 | 0.4 ± 0.1 | 0.8 ± 0.1 | 30 (88%) | 3.2 ± 1.6 | 0.7 ± 0.1 | 1.0 ± 0.0 | 33 (97%) |
| **3: HFD left (n=12)** | - | - | - | 0 (0%) | - | - | - | 0 (0%) | - | - | - | 0 (0%) | - | - | - | 0 (0%) |
| **4: HFS right (n=17)** | - | - | - | 0 (0%) | - | - | - | 0 (0%) | - | - | - | 0 (0%) | - | - | - | 0 (0%) |
| **5: HFS Rotated left (n=16)** | 25.7 ± 31.2 | 0.2 ± 0.1 | 0.6 ± 0.4 | 3 (19%) | 2.9 ± 1.2 | 0.5 ± 0.2 | 0.9 ± 0.1 | 3 (19%) | 12.8 ± 0.0 | 0.1 ± 0.0 | 0.5 ± 0.0 | 5 (31%) | 29.7 ± 19.7 | 0.2 ± 0.1 | 0.4 ± 0.4 | 3 (19%) |
| **6: HFS Rotated right (n=17)** | 3.0 ± 1.7 | 0.5 ± 0.3 | 0.8 ± 0.2 | 3 (18%) | 2.0 ± 0.0 | 0.6 ± 0.0 | 1.0 ± 0.0 | 2 (12%) | - | - | - | 0 (0%) | 22.7 ± 0.0 | 0.3 ± 0.0 | 0.6 ± 0.0 | 1 (6%) |
| **7: HFP left (n=7)** | - | - | - | 0 (0%) | - | - | - | 0 (0%) | - | - | - | 0 (0%) | - | - | - | 0 (0%) |
| **8: HFP right (n=3)** | - | - | - | 0 (0%) | - | - | - | 0 (0%) | - | - | - | 0 (0%) | - | - | - | 0 (0%) |
| **IOV** | - | - |  |  | - | - |  |  | - | 0.3 |  |  | - | 0.4 |  |  |
| **No of contours compared** | 58 | | | | 56 | | | | 55 | | | | 65 | | | |

| **OAR** | **Optic nerve R** | | | | **Parotid L** | | | | **Parotid R** | | | | **Spinal cord** | | | |
| --- | --- | --- | --- | --- | --- | --- | --- | --- | --- | --- | --- | --- | --- | --- | --- | --- |
| **Category** | **HD95** | **DSC** | **sDSC** | **n (%)** | **HD95** | **DSC** | **sDSC** | **n (%)** | **HD95** | **DSC** | **sDSC** | **n (%)** | **HD95** | **DSC** | **sDSC** | **n (%)** |
| **1: HFS straight (n=31)** | 3.4 ± 2.2 | 0.7 ± 0.1 | 1.0 ± 0.1 | 28 (90%) | 20.9 ± 42.9 | 0.7 ± 0.3 | 0.8 ± 0.3 | 8 (26%) | 5.1 ± 2.4 | 0.8 ± 0.1 | 0.9 ± 0.1 | 8 (26%) | 94.5 ± 34.9 | 0.5 ± 0.1 | 0.6 ± 0.2 | 23 (74%) |
| **2: HFS hyperextended (n=34)** | 3.8 ± 1.6 | 0.6 ± 0.1 | 1.0 ± 0.0 | 33 (97%) | 7.4 ± 11.1 | 0.9 ± 0.1 | 0.9 ± 0.1 | 15 (44%) | 6.6 ± 5.1 | 0.8 ± 0.1 | 0.9 ± 0.1 | 14 (41%) | 91.0 ± 36.6 | 0.4 ± 0.1 | 0.5 ± 0.2 | 21 (62%) |
| **3: HFD left (n=12)** | - | - | - | 0 (0%) | - | - | - | 0 (0%) | - | - | - | 0 (0%) | 89.8 ± 0.0 | 0.4 ± 0.0 | 0.5 ± 0.0 | 1 (8%) |
| **4: HFS right (n=17)** | - | - | - | 0 (0%) | - | - | - | 0 (0%) | - | - | - | 0 (0%) | 42.0 ± 34.1 | 0.5 ± 0.1 | 0.6 ± 0.1 | 6 (35%) |
| **5: HFS Rotated left (n=16)** | 12.4 ± 10.4 | 0.5 ± 0.3 | 0.7 ± 0.3 | 2 (13%) | 61.5 ± 73.3 | 0.5 ± 0.4 | 0.5 ± 0.4 | 3 (19%) | 21.8 ± 1.8 | 0.5 ± 0.3 | 0.6 ± 0.2 | 2 (13%) | 82.5 ± 26.1 | 0.6 ± 0.1 | 0.7 ± 0.1 | 15 (94%) |
| **6: HFS Rotated right (n=17)** | 39.2 ± 0.0 | 0.1 ± 0.0 | 0.3 ± 0.0 | 1 (6%) | 48.2 ± 50.4 | 0.3 ± 0.3 | 0.3 ± 0.3 | 4 (24%) | 20.2 ± 12.6 | 0.6 ± 0.1 | 0.6 ± 0.2 | 5 (29%) | 94.4 ± 45.3 | 0.5 ± 0.2 | 0.6 ± 0.2 | 13 (76%) |
| **7: HFP left (n=7)** | - | - | - | 0 (0%) | - | - | - | 0 (0%) | 108.0 ± 0.0 | 0.0 ± 0.0 | 0.0 ± 0.0 | 1 (14%) | 5.4 ± 2.6 | 0.8 ± 0.0 | 0.9 ± 0.0 | 2 (29%) |
| **8: HFP right (n=3)** | - | - | - | 0 (0%) | - | - | - | 0 (0%) | 100.9 ± 0.0 | 0.0 ± 0.0 | - | 1 (33%) | 159.1 ± 0.0 | 0.0 ± 0.0 | - | 1 (33%) |
| **IOV** | - | - | - | - | 4.9 | 0.8 | - | - | 5.1 | 0.8 |  |  | 12.1 | 0.8 |  |  |
| **No of contours compared** | 64 | | | | 30 | | | | 31 | | | | 82 | | | |

**Supplementary data B**

*Table with mean and standard deviation per organ at risk (OAR) for the pediatric subanalysis using the Dice Similarity Coefficient (DSC), Hausdorff Distance 95^th^ percentile (HD95), surface DSC (sDSC) and volume with the corresponding p-values from the Mann-Whitney U test. Only ROIs with at least three samples from both groups are analyzed. Level of significance was set at 0.05 divided by eleven as per Bonferroni correction for multiple testing. a = autocontour, DSC = dice similarity coefficient, HD95 = Hausdorff Distance, 95^th^ percentile, m = manual contour, L = left, R = right, SD = standard deviation.*

|  | **HD95 (mm, mean** ± **SD)** | | | **DSC (mean** ± **SD)** | | | **sDSC (mean** ± **SD)** | | | **Volume (cm^3^, mean** ± **SD)** | | | | | |
| --- | --- | --- | --- | --- | --- | --- | --- | --- | --- | --- | --- | --- | --- | --- | --- |
| **ROI** | **Adult scans** | **Pedi-atric scans** | **p-value** | **Adult scans** | **Pedi-atric scans** | **p-value** | **Adult scans** | **Pedi-atric scans** | **p-value** | **Adult scans** | | **Pediatric scans** | | **p-value** | |
|  |  |  |  |  |  |  |  |  |  | **m** | **a** | **m** | **a** | **m** | **a** |
| **Brain** | 2.2 ± 1.6 | 1.5 ± 0.5 | 0.05 | 1.0 ± 0.0 | 1.0 ± 0.0 | 0.02 | 1.0 ± 0.0 | 1.0 ± 0.0 | 0.51 | 1429.0 ± 160.8 | 1403.7 ± 158.9 | 1377.5 ± 147.7 | 1367.8 ± 158.9 | 0.66 | 0.73 |
| **Brainstem** | 6.3 ± 9.6 | 3.6 ± 1.1 | 0.16 | 0.9 ± 0.1 | 0.9 ± 0.0 | 0.10 | 0.9 ± 0.1 | 1.0 ± 0.0 | 0.12 | 27.5 ± 3.9 | 25.7 ± 3.0 | 24.7 ± 3.8 | 24.3 ± 3.5 | 0.10 | 0.16 |
| **Eye L** | 2.0 ± 2.0 | 1.7 ± 0.4 | 0.02 | 0.9 ± 0.1 | 0.9 ± 0.0 | 0.08 | 1.0 ± 0.1 | 1.0 ± 0.0 | 0.56 | 8.1 ± 1.0 | 7.9 ± 1.2 | 7.7 ± 1.1 | 8.2 ± 0.7 | 0.66 | 0.60 |
| **Eye R** | 2.8 ± 8.5 | 1.9 ± 0.3 | 0.04 | 0.9 ± 0.1 | 0.9 ± 0.0 | 0.05 | 1.0 ± 0.1 | 1.0 ± 0.0 | 0.40 | 8.3 ± 1.0 | 8.0 ± 1.4 | 7.9 ± 0.8 | 8.3 ± 0.5 | 0.31 | 0.56 |
| **Lens L** | 1.4 ± 0.6 | 1.1 ± 0.7 | 0.67 | 0.8 ± 0.1 | 0.8 ± 0.1 | 0.21 | 1.0 ± 0.0 | 1.0 ± 0.0 | 1.00 | 0.2 ± 0.1 | 0.2 ± 0.0 | 0.2 ± 0.0 | 0.1 ± 0.02 | 0.22 | 0.07 |
| **Lens R** | 1.3 ± 1.1 | 1.2 ± 0.6 | 0.04 | 0.8 ± 0.2 | 0.8 ± 0.1 | 0.33 | 1.0 ± 0.1 | 1.0 ± 0.0 | 0.73 | 0.2 ± 0.1 | 0.2 ± 0.0 | 0.2 ± 0.0 | 0.1 ± 0.0 | 0.14 | 0.01 |
| **Optic chiasm** | 8.7 ± 2.7 | 9.4 ± 6.9 | 0.43 | 0.4 ± 0.1 | 0.4 ± 0.3 | 0.39 | 0.8 ± 0.1 | 0.7 ± 0.3 | 0.47 | 0.5 ± 0.2 | 0.3 ± 0.0 | 0.5 ± 0.1 | 0.2 ± 0.1 | 0.77 | 0.08 |
| **Optic nerve L** | 3.1 ± 1.6 | 2.9 ± 1.4 | 0.81 | 0.7 ± 0.1 | 0.7 ± 0.1 | 0.07 | 1.0 ± 0.0 | 1.0 ± 0.0 | 0.56 | 0.7 ± 0.2 | 0.6 ± 0.0 | 0.6 ± 0.1 | 0.5 ± 0.1 | 0.48 | **<0.01** |
| **Optic nerve R** | 3.5 ± 1.8 | 4.5 ± 2.2 | 0.44 | 0.7 ± 0.1 | 0.6 ± 0.0 | 0.28 | 1.0 ± 0.0 | 1.0 ± 0.0 | 0.17 | 0.7 ± 0.2 | 0.6 ± 0.1 | 0.6 ± 0.1 | 0.5 ± 0.1 | 0.72 | **<0.01** |
| **Parotid L** | 11.4 ± 29.0 | 15.4 ± 19.2 | 0.56 | 0.8 ± 0.2 | 0.8 ± 0.1 | 0.33 | 0.9 ± 0.2 | 0.8 ± 0.2 | 1.00 | 28.9 ± 12.8 | 25.9 ± 9.9 | 10.5 ± 2.2 | 17.7 ± 9.1 | **<0.01** | 0.04 |
| **Parotid R** | 5.8 ± 3.2 | 7.1 ± 7.4 | 0.22 | 0.8 ± 0.1 | 0.8 ± 0.1 | 0.38 | 0.9 ± 0.1 | 0.9 ± 0.1 | 0.71 | 24.2 ± 7.7 | 24.7 ± 9.3 | 10.9 ± 2.6 | 16.4 ± 8.2 | **<0.01** | 0.02 |

**Supplementary data C**

*Table with mean and standard deviation per organ at risk (OAR) for the subanalysis of HFS straight and HFS hyperextended orientations using the Dice Similarity Coefficient (DSC), Hausdorff Distance 95^th^ percentile (HD95) and surface DSC (sDSC) with the corresponding p-values from the Mann-Whitney U test. Level of significance was set at 0.05 divided by eleven as per Bonferroni correction for multiple testing. DSC = dice similarity coefficient, HD95 = Hausdorff Distance, 95^th^ percentile, L = left, R = right, SD = standard deviation.*

|  | **HD95 (mean** ± **SD)** | | | **DSC (mean** ± **SD)** | | | | **Surface DSC (mean** ± **SD)** | | |
| --- | --- | --- | --- | --- | --- | --- | --- | --- | --- | --- |
| **OAR** | **HFS straight scans** | **HFS hyperextended scans** | **p-value** | **HFS straight scans** | **HFS hyperextended scans** | **p-value** | **HFS straight scans** | | **HFS hyperextended scans** | **p-value** |
| **Mandible** | 10.1 ± 3.0 | 7.0 ± 3.0 | 0.01 | 0.8 ± 0.0 | 0.8 ± 0.1 | 0.25 | 0.9 ± 0.0 | | 0.9 ± 0.0 | 0.75 |
| **Brain** | 2.1 ± 0.5 | 2.4 ± 2.2 | 0.64 | 1.0 ± 0.0 | 1.0 ± 0.0 | 0.50 | 1.0 ± 0.0 | | 1.0 ± 0.0 | 0.03 |
| **Brainstem** | 4.2 ± 2.0 | 5.8 ± 3.3 | 0.03 | 0.9 ± 0.1 | 0.9 ± 0.0 | 0.87 | 0.9 ± 0.1 | | 0.9 ± 0.0 | 0.24 |
| **Oral cavity** | 17.0 ± 7.3 | 16.1 ± 6.4 | 0.95 | 0.7 ± 0.1 | 0.7 ± 0.1 | 0.95 | 0.6 ± 0.1 | | 0.6 ± 0.1 | 0.87 |
| **Eye L** | 2.3 ± 2.8 | 1.6 ± 0.5 | 0.19 | 0.9 ± 0.1 | 0.9 ± 0.0 | 0.14 | 1.0 ± 0.1 | | 1.0 ± 0.0 | 0.08 |
| **Eye R** | 4.2 ± 12.2 | 1.6 ± 0.7 | 0.75 | 0.9 ± 0.2 | 0.9 ± 0.0 | 0.95 | 1.0 ± 0.2 | | 1.0 ± 0.0 | 0.78 |
| **Lacrimal gland L** | 8.8 ± 4.4 | 7.7 ± 4.1 | 0.35 | 0.2 ± 0.2 | 0.3 ± 0.2 | 0.16 | 0.6 ± 0.3 | | 0.7 ± 0.3 | 0.21 |
| **Lacrimal gland R** | 8.1 ± 4.4 | 8.5 ± 4.6 | 0.86 | 0.3 ± 0.2 | 0.4 ± 0.2 | 0.15 | 0.6 ± 0.3 | | 0.7 ± 0.3 | 0.40 |
| **Lens L** | 1.4 ± 0.7 | 1.3 ± 0.6 | 0.83 | 0.8 ± 0.1 | 0.8 ± 0.1 | 0.69 | 1.0 ± 0.0 | | 1.0 ± 0.0 | 1.00 |
| **Lens R** | 1.5 ± 1.4 | 1.2 ± 0.6 | 0.73 | 0.8 ± 0.2 | 0.8 ± 0.1 | 0.63 | 1.0 ± 0.1 | | 1.0 ± 0.0 | 0.27 |
| **Optic chiasm** | 8.2 ± 3.3 | 9.1 ± 2.0 | 0.01 | 0.4 ± 0.2 | 0.4 ± 0.1 | 0.41 | 0.8 ± 0.1 | | 0.8 ± 0.1 | 0.21 |
| **Optic nerve L** | 2.9 ± 1.4 | 3.3 ± 1.6 | 0.16 | 0.7 ± 0.1 | 0.7 ± 0.1 | 0.09 | 1.0 ± 0.0 | | 1.0 ± 0.0 | 0.39 |
| **Optic nerve R** | 3.2 ± 2.0 | 3.8 ± 1.6 | 0.05 | 0.7 ± 0.1 | 0.7 ± 0.1 | 0.17 | 1.0 ± 0.1 | | 1.0 ± 0.0 | 0.07 |
| **Parotid L** | 22.6 ± 45.6 | 4.9 ± 1.9 | 0.83 | 0.8 ± 0.3 | 0.9 ± 0.0 | 0.97 | 0.8 ± 0.3 | | 0.9 ± 0.1 | 0.97 |
| **Parotid R** | 4.9 ± 2.5 | 6.3 ± 3.5 | 0.42 | 0.8 ± 0.1 | 0.8 ± 0.1 | 0.66 | 0.9 ± 0.1 | | 0.9 ± 0.1 | 1.00 |
| **Spinal cord** | 96.5 ± 34.3 | 98.7 ± 33.0 | 0.67 | 0.5 ± 0.1 | 0.4 ± 0.1 | 0.34 | 0.6 ± 0.2 | | 0.5 ± 0.2 | 0.44 |

**Supplementary data D**

Distribution of scores for all organs at risk (OARs) in the qualitative analysis with the median score of each OAR, separated by positioning category. a) Qualitative scoring for HFS straight position. b) Qualitative scoring for HFS hyperextended position. L = left, R = right.


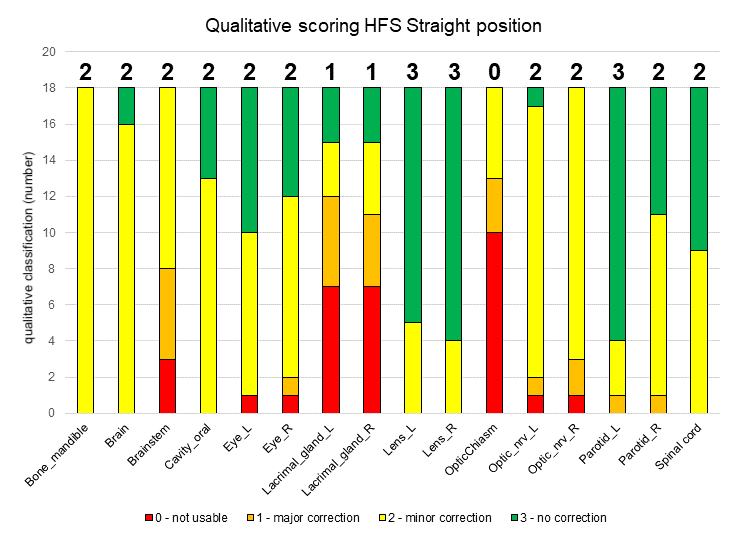


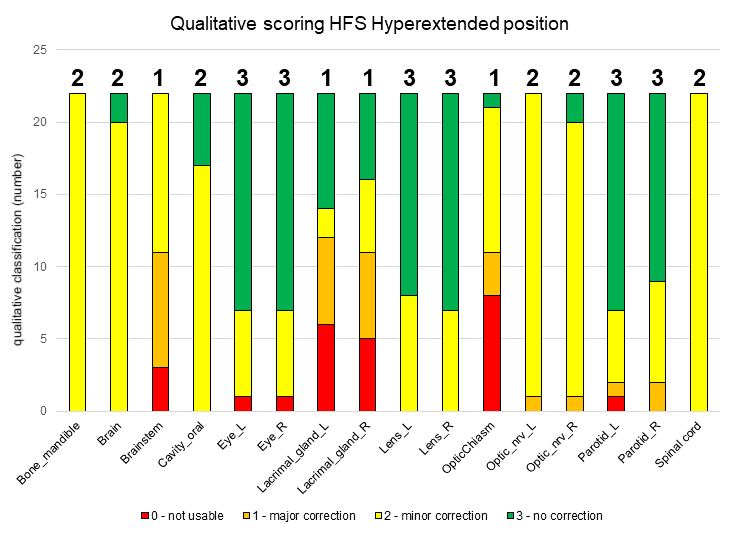


**Supplementary data E**

Table with median, range and p-values from Wilcoxon test for D_2%_ [Gy (RBE)] and D_mean_ [Gy (RBE)] of all organs at risk (OAR). Threshold for significance was set at 0.05 divided by sixteen (number of analyzed OARs) as per Bonferroni correction for multiple testing.

| **OAR** | **D_2%_ manual contours [median, range] [Gy (RBE)]** | **D_2%_ autocontours [median, range] [Gy (RBE)]** | **p-value Wilcoxon test** | **D_mean_ manual contours [median, range] [Gy (RBE)]** | **D_mean_ autocontours [median, range] [Gy (RBE)]** | **p-value Wilcoxon test** |
| --- | --- | --- | --- | --- | --- | --- |
| **Mandible** | 27.7 [0.0 – 58.1] | 24.0 [0.0 – 51.6] | <0.01 | 3.0 [0.0 – 9.3] | 2.4 [0.0 – 9.7] | **<0.01** |
| **Brain** | 36.1 [1.1-64.0] | 35.3 [0.9 – 63.4] | 0.83 | 3.5 [0.1 – 13.1] | 3.5 [0.1 – 13.0] | 0.47 |
| **Brainstem** | 24.9 [0.2 – 59.4] | 25.5 [0.2 – 60.5] | **<0.01** | 9.1 [0.0 – 31.1] | 9.4 [0.0 – 33.9] | **<0.01** |
| **Oral cavity** | 18.4 [0.1 – 58.7] | 22.0 [0.0 – 57.5] | <0.01 | 1.9 [0.0 – 15.4] | 1.8 [0.0 – 15.7] | <0.01 |
| **Eye L** | 8.2 [0.0 – 38.4] | 7.9 [0.0 – 39.2] | 0.85 | 1.9 [0.0 – 11.1] | 2.1 [0.0 – 11.3] | 0.11 |
| **Eye R** | 12.2 [0.0 – 43.2] | 11.7 [0.0 – 44.2] | 0.47 | 2.1 [0.0 – 14.8] | 2.1 [0.0 – 16.8] | 0.99 |
| **Lacrimal gland L** | 3.8 [0.0 – 20.0] | 1.6 [0.0 – 23.2] | 0.04 | 1.4 [0.0 – 8.2] | 0.6 [0.0 – 6.6] | 0.11 |
| **Lacrimal gland R** | 8.7 [0.0 – 42.6] | 4.2 [0.0 – 36.7] | 0.04 | 2.7 [0.0 – 29.8] | 1.0 [0.0 – 21.5] | 0.07 |
| **Lens L** | 0.9 [0.0 – 10.9] | 0.8 [0.0 – 10.7] | 0.01 | 0.6 [0.0 – 5.6] | 0.6 [0.0 – 5.2] | 0.11 |
| **Lens R** | 0.6 [0.0 – 14.4] | 0.4 [0.0 – 14.3] | 0.86 | 0.2 [0.0 – 7.4] | 0.2 [0.0 – 7.5] | 0.69 |
| **Optic chiasm** | 24.8 [0.1 – 53.9] | 24.2 [0.1 – 54.4] | 0.45 | 20.7 [0.0 – 49.4] | 21.9 [0.0 – 53.7] | **<0.01** |
| **Optic nerve L** | 31.7 [0.0 – 53.1] | 31.6 [0.0 – 54.0] | **<0.01** | 17.8 [0.0 – 36.4] | 19.3 [0.0 – 42.3] | **<0.01** |
| **Optic nerve R** | 35.3 [0.1 – 56.9] | 36.4 [0.1 – 56.7] | 0.06 | 20.5 [0.0 – 45.2] | 22.0 [0.0 – 51.7] | **<0.01** |
| **Parotid L** | 12.2 [0.2 – 50.6] | 9.4 [0.0 – 48.1] | 0.27 | 2.0 [0.0 – 26.2] | 2.0 [0.0 – 25.9] | 0.31 |
| **Parotid R** | 17.2 [0.0 – 50.8] | 17.4 [0.0 – 55.4] | 0.13 | 1.7 [0.0 – 35.0] | 3.2 [0.0 – 35.3] | 0.22 |
| **Spinal cord** | 0.5 [0.0 – 21.4] | 0.4 [0.0 – 21.2] | 0.65 | 0.1 [0.0 – 9.4] | 0.1 [0.0 – 3.7] | 0.01 |

**Supplementary data F**

Table of median, range and p-values from Mann Whitney U test for D2% [Gy (RBE)] and Dmean [Gy (RBE)] of all ROIs, comparison between HFS straight and HFS hyperextended orientations. Threshold for significance was set at 0.05 divided by 32 (number of analyzed organs at risk) as per Bonferroni correction for multiple testing.

| **ROIs** | **D_2%_ contours in HFS straight [median, range] [Gy (RBE)]** | **D_2%_ contours in HFS hyperextended [median, range] [Gy (RBE)]** | **p-value Mann Whitney U test** | **D_mean_ contours in HFS straight [median, range] [Gy (RBE)]** | **D_mean_ contours in HFS hyperextended [median, range] [Gy (RBE)]** | **p-value Mann Whitney U test** |
| --- | --- | --- | --- | --- | --- | --- |
| ***Manual contours*** |  |  |  |  |  |  |
| **Mandible** | 28.9 [0.0 - 58.1] | 27.4 [5.5 - 36.7] | 0.61 | 2.2 [0.0 - 8.6] | 3.4 [0.6 - 9.3] | 0.76 |
| **Brain** | 50.1 [1.1 – 64.0] | 30.7 [6.9 - 49.0] | 0.11 | 4.8 [0.1 - 13.1] | 3.2 [0.5 - 8.2] | 0.31 |
| **Brainstem** | 47.1 [0.2 - 59.4] | 24.1 [0.5 - 54.4] | 0.54 | 15.3 [0.0 - 31.1] | 5.1 [0.0 - 22.0] | 0.54 |
| **Oral cavity** | 4.8 [0.1 - 18.4] | 37.4 [9.2 - 58.7] | 0.03 | 0.4 [0.0 - 1.9] | 8.4 [1.0 - 15.4] | 0.03 |
| **Eye L** | 5.2 [0.0 - 10.2] | 16.0 [0.0 - 38.4] | 0.02 | 0.7 [0.0 - 2.9] | 4.5 [0.0 - 11.2] | 0.01 |
| **Eye R** | 2.6 [0.0 - 26.5] | 18.3 [0.0 - 43.2] | 0.03 | 0.5 [0.0 - 6.8] | 5.2 [0.0 - 14.8] | 0.03 |
| **Lacrimal gland L** | 0.3 [0.0 - 4.4] | 9.8 [2.1 – 20.0] | <0.01 | 0.1 [0.0 - 1.4] | 3.8 [0.5 - 8.2] | <0.01 |
| **Lacrimal gland R** | 2.3 [0.0 - 26.5] | 16.3 [3.1 - 42.6] | 0.05 | 0.9 [0.0 - 15.7] | 6.0 [1.2 - 29.8] | 0.03 |
| **Lens L** | 0.1 [0.0 - 6.1] | 2.5 [0.0 - 10.9] | 0.06 | 0.1 [0.0 - 2.5] | 1.1 [0.0 - 5.6] | 0.03 |
| **Lens R** | 0.2 [0.0 - 2.1] | 2.7 [0.0 - 14.4] | 0.01 | 0.1 [0.00 – 1.0] | 1.8 [0.0 - 7.4] | 0.01 |
| **Optic chiasm** | 52.5 [0.1 - 53.9] | 23.6 [7.1 - 50.5] | 0.15 | 39.7 [0.0 - 49.4] | 17.7 [1.4 – 39.0] | 0.11 |
| **Optic nerve L** | 48.8 [0.0 - 53.1] | 23.9 [9.9 - 52.6] | 0.15 | 18.2 [0.0 - 34.4] | 17.3 [4.6 - 36.4] | 0.88 |
| **Optic nerve R** | 52.7 [0.1 - 56.9] | 26.9 [6.8 - 55.0] | 0.17 | 22.3 [0.0 - 45.2] | 19.1 [4.4 - 43.6] | 0.88 |
| **Parotid L** | 10.6 [1.7 - 22.6] | 12.2 [0.2 - 50.6] | 0.83 | 1.1 [0.1 - 14.1] | 3.4 [0.0 - 26.2] | 0.60 |
| **Parotid R** | 23.6 [0.0 - 39.4] | 17.2 [0.1 - 50.8] | 1.00 | 4.1 [0.0 – 35.0] | 1.7 [0.0 - 24.0] | 1.00 |
| **Spinal cord** | 0.5 [0.0 - 21.4] | 0.5 [0.0 - 20.6] | 0.80 | 0.1 [0.0 - 9.4] | 0.1 [0.0 - 4.5] | 0.57 |
| ***Autocontours*** |  |  |  |  |  |  |
| **Mandible** | 19.4 [0.0 - 51.6] | 25.7 [0.0 - 35.9] | 1.00 | 1.6 [0.0 - 9.7] | 2.7 [0.0 - 8.2] | 0.60 |
| **Brain** | 52.9 [0.9 - 63.4] | 28.2 [6.9 - 49.7] | 0.08 | 5.4 [0.1 – 13.0] | 2.9 [0.5 - 8.2] | 0.25 |
| **Brainstem** | 49.0 [0.2 - 60.5] | 24.2 [0.5 - 54.8] | 0.54 | 17.9 [0.0 - 33.9] | 5.3 [0.1 - 21.9] | 0.45 |
| **Oral cavity** | 0.9 [0.0 - 37.1] | 32.0 [0.0 - 57.5] | 0.04 | 0.1 [0.0 - 15.7] | 5.4 [0.0 - 12.3] | 0.05 |
| **Eye L** | 3.4 [0.0 - 9.8] | 18.2 [0.0 - 39.2] | 0.01 | 0.5 [0.0 - 2.7] | 5.2 [0.0 - 11.3] | 0.01 |
| **Eye R** | 2.6 [0.0 - 25.3] | 18.8 [0.0 - 44.2] | 0.04 | 0.4 [0.0 - 6.3] | 5.2 [0.0 - 16.8] | 0.03 |
| **Lacrimal gland L** | 0.4 [0.0 - 2.6] | 2.8 [0.0 - 23.2] | 0.01 | 0.1 [0.0 - 1.5] | 1.5 [0.0 - 6.6] | 0.01 |
| **Lacrimal gland R** | 0.6 [0.0 - 16.2] | 8.0 [0.0 - 36.7] | 0.05 | 0.3 [0.0 - 6.9] | 3.0 [0.0 - 21.5] | 0.07 |
| **Lens L** | 0.1 [0.0 - 5.4] | 2.4 [0.0 - 10.7] | 0.06 | 0.1 [0.0 - 2.6] | 1.0 [0.0 - 5.2] | 0.03 |
| **Lens R** | 0.2 [0.0 - 2.3] | 2.7 [0.0 - 14.3] | 0.01 | 0.1 [0.0 - 1.0] | 1.8 [0.0 - 7.5] | 0.01 |
| **Optic chiasm** | 52.5 [0.1 - 54.4] | 23.8 [5.0 - 51.5] | 0.20 | 43.0 [0.0 - 53.7] | 19.3 [3.2 - 45.3] | 0.17 |
| **Optic nerve L** | 52.2 [0.0 - 54.0] | 25.4 [10.3 - 53.2] | 0.17 | 21.8 [0.0 - 42.3] | 18.6 [6.2 - 40.1] | 0.76 |
| **Optic nerve R** | 53.6 [0.1 - 56.7] | 26.9 [6.3 – 55.0] | 0.15 | 24.9 [0.0 - 51.7] | 19.6 [4.0 - 47.7] | 0.76 |
| **Parotid L** | 4.0 [0.0 - 41.2] | 12.1 [0.1 - 48.1] | 0.36 | 0.5 [0.0 - 14.1] | 2.7 [0.0 - 25.9] | 0.22 |
| **Parotid R** | 11.1 [0.0 - 55.4] | 17.5 [0.1 - 47.3] | 0.94 | 3.2 [0.0 - 35.3] | 3.2 [0.0 - 23.9] | 0.94 |
| **Spinal cord** | 0.5 [0.0 - 15.6] | 0.3 [0.0 - 21.2] | 0.54 | 0.0 [0.0 - 2.7] | 0.1 [0.0 - 3.7] | 0.32 |
